# Genome sequence of *Microbacterium* sp. MAHUQ-60 isolated from the rhizosphere of eggplant located in Anseong, South Korea

**DOI:** 10.1128/mra.01132-25

**Published:** 2025-12-04

**Authors:** Md. Amdadul Huq, Muhammad Zubair Siddiqi, M. Mizanur Rahman

**Affiliations:** 1 Department of Life Sciences, College of BioNano Technology, Gachon University, Seongnam-si, Gyeonggi-do, Republic of Korea; 2 Department of Biotechnology, Hankyong National University 105909, Anseong-si, Gyeonggi-do, Republic of Korea; 3 Department of Biotechnology and Genetic Engineering, Faculty of Biological Science, Islamic University 247297, Kushtia, Bangladesh; University of Manitoba, Winnipeg, Manitoba, Canada

**Keywords:** genome sequence, *Microbacterium *sp. MAHUQ-60, eggplant

## Abstract

This study reports the draft genome sequence of *Microbacterium* sp. MAHUQ-60, a bacterial strain isolated from the rhizosphere of an eggplant located in Anseong, South Korea, in 2019. The genome consists of 3,425,239 base pairs assembled into five contigs, encoding 3,194 predicted protein coding genes.

## ANNOUNCEMENT

The genus *Microbacterium*, which was first established by Orla-Jensen (1), belongs to the family *Microbacteriaceae* and currently comprises 166 validly published species (2) that have been isolated from a large variety of sources (3–5). During investigations on bacterial biodiversity in the rhizosphere of an eggplant, a new strain of the genus *Microbacterium*, designated *Microbacterium* sp. MAHUQ-60, was isolated, and its draft genome assembly is presented in this study. Sequencing bacterial genomes serves as a valuable technique with broad applications in medicine, public health, and evolutionary research (6–10).

Strain MAHUQ-60 was isolated from the rhizosphere of an eggplant located in Anseong, South Korea. Soil samples were collected from the rhizosphere by gently removing soil adhering to the plant roots. One gram of soil sample was suspended in 9 mL of sterile 0.85% (wt/vol) NaCl solution. The suspension was serially diluted up to a 10^−6^ dilution, and 100 µL of individual dilution was spread onto nutrient agar (NA; Difco, BD, Cat. No. 213000) plates. Then, the plates were placed in an incubator at 30°C for 3 days under aerobic conditions. Single colonies were purified by repeated streaking on fresh NA plates. Strain MAHUQ-60 was identified as *Microbacterium* sp. MAHUQ-60 based on 16S rRNA gene sequence analysis. MAHUQ-60 has been deposited to China General Microbiological Culture Collection Center with deposition number CGMCC 1.60115. The genomic DNA was extracted from the bacterial culture that was grown in nutrient broth (Difco, BD, Cat. No. 234000) for 24 hours using a genomic DNA extraction kit according to the manufacturer’s protocol (Solgent, Republic of Korea). The DNA fragments were size selected using AMPure XP beads to isolate those within the desired range, typically 200–300 base pairs (bp) for standard Illumina sequencing libraries. The library for sequencing was prepared using the Nextera XT DNA Library Prep Kit (Illumina, San Diego, USA). After library preparation, sequencing was carried out using the Illumina HiSeq 3000 platform with paired-end 2 × 150 bp reads following the standard protocol provided by the manufacturer. Reads were quality-controlled using FastQC (11), and adapter sequences and low-quality bases were trimmed with Trimmomatic (12). Illumina’s bcl2fastq software v.2.20.0 was utilized with default parameters to demultiplex the raw reads and convert them into FASTQ format. Reads were processed with Trimmomatic v.0.38 (12). The genome sequence was assembled by SOAPdenovo v.2.04 assembler (13). The genome was annotated with the NCBI Prokaryotic Genome Annotation Pipeline v.6.3 (14–16). The strain was identified using a whole genome-based taxonomic analysis in the Type (Strain) Genome Server (https://tygs.dsmz.de). The Genome-to-Genome Distance bioinformatics tool (17) was used to measure *in silico* DNA-DNA hybridization values. The best match to the strain MAHUQ-60 genome is the type strain *Microbacterium suwonense* NBRC 106310 (accession number GCA_030296555) with *in silico* DNA-DNA hybridization values of 25.7%.

The genome of *Microbacterium* sp. MAHUQ-60 is 3,425,239 bp long with a GC content of 69.0%. The assembly contains five contigs with a coverage of 112×. A total of 3,253 genes were predicted, of which 3,194 are protein-coding genes. The details of genomic features are presented in Table 1.

**TABLE 1:** The genomic data, assembly, annotation, and source information of strain MAHUQ-60

Feature	Result for strain MAHUQ-60
Description of source	
Location	Anseong, South Korea
Time	2019
Type	Rhizosphere of eggplant
Sequencing summary	
Coverage	112×
Genome size (bp)	3,425,239
GC content (%)	69.0
Assembly report	
Contigs	5
Contig *L*_50_	2
Contig *N*_50_	1.1 Mb
Annotation report	
Genes (total)	3,253
CDSs (total)	3,198
CDSs (with protein)	3,194
ncRNAs	3
tRNA	46
Quality analysis	
Completeness (%)	98.75
Contamination (%)	2.73

## Data Availability

The draft genome sequence of *Microbacterium* sp. MAHUQ-60 was deposited in GenBank under accession number JBRFIK000000000. The raw reads are available in SRA under accession number SRR33604966. The version described in this paper is the first version, JBRFIK000000000.1.
